# Combined clinical and DWI radiomics model for predicting 90-day functional outcomes after intravenous thrombolysis in acute ischemic stroke patients

**DOI:** 10.3389/fneur.2026.1847290

**Published:** 2026-08-28

**Authors:** Yupeng Bai, Qifeng Liu, Ruonan Zhan, Qiaoqiao Xu, Chunhua Xia, Yinfeng Qian

**Affiliations:** 1 Department of Radiology, The First Affiliated Hospital of Anhui Medical University, Hefei, Anhui, China; 2 Medical Image Center, The Third Affiliated Hospital of Anhui Medical University Hefei, Anhui, China

**Keywords:** acute ischemic stroke, diffusion-weighted imaging, intravenous thrombolysis, mRS scores, radiomics model

## Abstract

**Objective:**

To develop an interpretable machine learning model based on Diffusion-Weighted Imaging (DWI) radiomics and D-dimer for 90-day outcome prediction after intravenous thrombolysis in acute ischemic stroke (AIS), and to explore candidate upstream transcriptional programs of D-dimer using publicly available rat middle cerebral artery occlusion (MCAO) datasets as an exploratory, hypothesis-generating step.

**Methods:**

This retrospective study included 115 AIS patients treated with intravenous thrombolysis (January 2024–September 2025). Patients were stratified by 90-day modified Rankin Scale (mRS) scores into favorable (0–2) and unfavorable (1–4) outcome groups. DWI radiomic features were extracted and selected to construct four machine learning models (Logistic Regression, Decision Tree, LDA, LightGBM). Independent clinical risk factors were identified to build a clinical model. The best machine learning model was combined with clinical factors to create a combined model (Combined-LR), interpreted via SHAP. Additionally, transcriptomic analysis of two rat MCAO datasets was conducted to investigate D-dimer-related molecular mechanisms.

**Results:**

The Radiomics-LR model showed superior performance (training AUC 0.926, testing AUC 0.857). D-dimer was an independent risk factor. Combined-LR achieved the best performance (training AUC 0.939, testing AUC 0.862; optimism-corrected AUC 0.838 by bootstrap, 0.772 by cross-validation), significantly outperforming the clinical model (training *P* < 0.001, testing *P* = 0.036). Decision curve analysis indicated that the Combined-LR provided higher net clinical benefit across multiple risk threshold intervals, suggesting greater practical utility for clinical prognostication. Transcriptomic analysis revealed upregulation of complement/coagulation genes, including *Serpine1* (PAI-1), *C5ar1*, and *Itgam* (CD11b).

**Conclusion:**

We established an interpretable machine learning model integrating DWI radiomics and clinical variables for predicting 90-day post-thrombolysis outcomes in AIS. Transcriptomic analyses provided hypothesis-generating insights into D-dimer-related mechanisms. External multi-center validation is required.

## 1 Introduction

Acute ischemic stroke (AIS) represents the leading cause of disability and mortality among Chinese residents, accounting for 80% of all cerebrovascular events (5, 6). Intravenous thrombolysis with tissue plasminogen activator (tPA) remains the cornerstone of acute-phase AIS management. However, clinical evidence demonstrates that 40%−60% of patients exhibit unfavorable functional outcomes at 90 days post-thrombolysis [modified Rankin Scale (mRS) score ≥3] (1, 2). The accurate identification of high-risk patients prior to treatment, enabling precise risk stratification and individualized therapeutic decisions, has emerged as a critical challenge in contemporary stroke care.

Current prognostic assessment in ischemic stroke predominantly relies on conventional scoring systems (e.g., National Institutes of Health Stroke Scale, NIHSS) and subjective radiological interpretation, which suffer from limited predictive accuracy and inter-observer variability (3, 4, 7). Although DWI-based radiomics integrated with machine learning can quantify lesion heterogeneity and enhance prognostication, clinical adoption remains hindered by opaque “black-box” models (8). Interpretable frameworks like SHAP (Shapley Additive Explanations) address this barrier by visualizing feature contributions, thereby transforming complex predictions into clinically comprehensible decision-support tools (9, 10).

To advance beyond existing limitations, this study introduces three core innovations. First, we systematically integrate DWI radiomics with clinical biomarkers (notably D-dimer) into a unified predictive model, overcoming the fragmentation of prior studies that relied on either pure imaging or pure clinical variables (11). Second, we employ SHAP-based interpretable machine learning with clinical net benefit validation, moving beyond traditional black-box algorithms to provide transparent, clinically actionable risk stratification (12). Third, we bridge the gap between predictive modeling and biological mechanism by exploring the molecular underpinnings of key predictors such as D-dimer, offering a biological rationale for clinical interventions rather than mere statistical association. We acknowledge that this is a single-center exploratory study; radiomic features are sensitive to scanner and protocol variations, and external multicenter validation is warranted before clinical implementation.

The present study therefore aimed to develop and compare machine learning models for 90-day functional outcome after intravenous thrombolysis using DWI radiomic features together with admission clinical variables, to interpret the selected model with SHAP and to assess its net clinical benefit by decision curve analysis.

## 2 Materials and methods

### 2.1 Patient characteristics

This retrospective study enrolled AIS patients admitted to the Department of Neurology at our hospital between January 2024 and September 2025. All patients received intravenous thrombolysis within 4.5 h of symptom onset. Brain MRI examination, including the DWI sequence, was performed within 24 h after thrombolysis. Inclusion criteria comprised: diagnosis of AIS according to the 2018 Chinese Guidelines for the Diagnosis and Treatment of Acute Ischemic Stroke; treatment with intravenous thrombolysis; completion of DWI sequence MRI scanning following thrombolysis; availability of complete clinical follow-up data and 90-day modified Rankin Scale (mRS) scores. Exclusion criteria included: non-ischemic lesions such as intracerebral hemorrhage, brain tumors, or traumatic brain injury; presence of severe image artifacts or poor image quality prior to thrombolysis; missing follow-up data or stroke-unrelated mortality; comorbid severe systemic diseases affecting prognostic assessment.

A total of 115 patients were ultimately enrolled. Using a random number table, the cohort was divided into training (*n* = 81) and testing (*n* = 34) sets at a 7:3 ratio. This study received approval from the Hospital Ethics Committee (approval number: 2025-271-01). The requirement for written informed consent was waived by the Ethics Committee. All data were anonymized in accordance with the Declaration of Helsinki.

### 2.2 Clinical and imaging data collection

Clinical data for all enrolled patients were collected using standardized case report forms by trained physicians. Baseline demographic information included sex and age (recorded in years). Stroke-related risk factors encompassed history of hypertension (defined as previously diagnosed hypertension or admission blood pressure ≥140/90 mmHg), alcohol consumption (defined as alcohol intake ≥1 time per week for more than 1 year), smoking (defined as cumulative cigarette consumption ≥100 cigarettes or cessation < 1 year), and prior stroke. Neurological deficit severity was assessed using the NIHSS at admission, performed by neurologists with NIHSS certification. Onset-to-treatment (OTT) time, defined as the interval from symptom onset to initiation of intravenous thrombolysis, was documented in minutes. No clinical or laboratory variable had missing values; all 115 patients had complete data for every variable included in the analysis.

Laboratory investigations included complete blood count, comprehensive metabolic panel, coagulation profile, and D-dimer levels, all completed within 4 h of admission. D-dimer was abstracted from the clinical record as a dichotomous result, normal or elevated, against the upper limit of the laboratory reference interval of 0.5 mg/L; the underlying concentrations were not entered into the study database and could not be recovered retrospectively. The 0.5 mg/L threshold was therefore adopted from the laboratory reference interval and was not derived from the present cohort, and no cohort-specific cut-point could be estimated. This is the form in which the result reaches the treating clinician at our institution, so the model uses D-dimer as it is actually reported in practice. Blood samples were drawn within 4 h of admission and, in all cases, before the administration of alteplase. D-dimer was measured by immunoturbidimetric assay on a Sysmex analyser with the Sysmex D-Dimer reagent kit; the laboratory reference interval is < 0.5 mg/L and the intra-assay coefficient of variation was < 5%. The primary outcome measure was functional status at 90 days, assessed using the modified Rankin Scale (mRS). Follow-up was conducted by trained personnel certified in mRS assessment through structured telephone interviews or outpatient visits. Telephone follow-up employed standardized questionnaires evaluating key indicators including activities of daily living and functional independence. Patients unable to complete telephone follow-up were required to attend in-person clinic evaluation. The follow-up window was set at 90 ± 7 days, with at least three contact attempts at different time periods for patients lost to follow-up. Based on mRS scores, patients were categorized into favorable outcome (mRS 0–2, functionally independent in daily activities) and unfavorable outcome (mRS 3–6, moderate to severe disability or death) groups.

### 2.3 MRI acquisition protocol

All MRI examinations were performed within 24 h after thrombolysis using a Philips Achieva 3.0T superconducting MRI scanner (Philips Healthcare, Netherlands) equipped with an 8-channel combined head-neck coil. Prior to scanning, patients underwent standardized preparation procedures, including removal of metallic objects, positioning (supine, head-first entry), and head immobilization to minimize motion artifacts.

DWI sequences were acquired using single-shot echo-planar imaging (SE-EPI) with the following parameters: echo time (TE) = 80 ms, repetition time (TR) = 3,600 ms, slice thickness = 5 mm, interslice gap = 1 mm, field of view (FOV) = 240 mm × 240 mm, acquisition matrix = 128 × 128, reconstruction matrix = 256 × 256, number of excitations (NEX) = 2. Diffusion-sensitizing gradients were applied along three orthogonal directions with b-values of 0 and 1,000 s/mm^2^. Axial DWI sequences covering the entire brain were obtained, yielding 24 slices per examination.

Image quality control measures included: real-time monitoring of head motion with immediate re-scanning upon detection of significant artifacts; assessment of image quality by experienced radiologic technologists, evaluating signal-to-noise ratio, contrast, and anatomical clarity; storage of all imaging data in DICOM format with establishment of a dedicated imaging database for centralized management. Images demonstrating severe motion artifacts, susceptibility artifacts, or poor quality due to technical failure were excluded from analysis.

### 2.4 Image processing and feature extraction

All DWI images underwent standardized preprocessing to ensure data consistency. The preprocessing workflow comprised: format conversion of DICOM images to NIfTI format for subsequent processing; resampling of all images to uniform voxel dimensions (1 × 1 × 1 mm^3^) using trilinear interpolation to eliminate inter-scan parameter variability; intensity normalization via *Z*-score standardization (μ = 0, σ = 1) to minimize systematic differences across scanning devices; intra-subject image registration using individual T1-weighted images as templates to ensure anatomical correspondence accuracy.

Region of interest (ROI) delineation of ischemic lesions was performed manually using ITK-SNAP software (version 3.8.0). Two experienced neuroradiologists, blinded to all clinical data and 90-day outcomes, independently segmented the lesion boundaries. Segmentation strictly avoided sulci, ventricles, and major vessels. In cases of discrepancy, a third senior neuroradiologist with over 10 years of experience made the final decision, adjudicating based on DWI core signal with apparent diffusion coefficient confirmation while excluding chronic lesions.

To assess feature extraction stability, a reproducibility analysis was conducted on 40 randomly selected patients. One month after the initial delineation, the same physician repeated the segmentation (intra-observer), and an additional independent physician performed a new segmentation (inter-observer). Intra- and inter-class correlation coefficients (ICCs) were calculated, with values > 0.75 considered indicative of good reproducibility. The analysis demonstrated excellent consistency: intra-observer ICCs were 0.963 and 0.951, while inter-observer ICCs ranged from 0.928 to 0.941. Specifically for lesion volume, the intra-reader ICC was 0.963 (95% CI 0.935–0.979) and the inter-reader ICC was 0.928 (95% CI 0.892–0.954).

Following image standardization, radiomic features were extracted using the PyRadiomics toolkit. A total of 1,024 features were obtained, encompassing multiple dimensions including gray-level co-occurrence matrix (GLCM), gray-level run-length matrix (GLRLM), shape, and intensity parameters. Feature selection and dimensionality reduction were subsequently conducted using *t*-tests, Pearson correlation coefficients, minimum redundancy maximum relevance (mRMR), and least absolute shrinkage and selection operator (LASSO) regression.

### 2.5 Model development and evaluation

High-dimensional radiomic features were extracted across seven categories: shape features describing geometric properties of ROIs, including volume, surface area, sphericity, among 14 parameters; first-order statistical features reflecting voxel intensity distribution within ROIs, encompassing mean, variance, skewness, kurtosis, among 18 parameters; GLCM quantifying spatial relationships between adjacent voxels, yielding 24 features; gray-level dependence matrix (GLDM) characterizing dependence relationships among voxels with identical gray values, extracting 14 features; gray-level run-length matrix (GLRLM) quantifying length distributions of consecutive identical gray values, generating 16 features; gray-level size zone matrix (GLSZM) describing size distributions of connected regions, producing 16 features; neighborhood gray-tone difference matrix (NGTDM) evaluating differences between central voxels and their neighborhoods, yielding 5 features.

Six prediction models were developed in the training cohort: logistic regression (Radiomics-LR), decision tree (DT), linear discriminant analysis (LDA), LightGBM, a clinical-only model incorporating exclusively clinical variables, and a combined model integrating both clinical and radiomic features. Model outputs represented the probability of unfavorable outcome classification. Model performance was evaluated in the testing cohort using area under the receiver operating characteristic curve, accuracy, sensitivity, and specificity. DeLong's test was employed to compare AUC values between models. Calibration curves assessed model goodness-of-fit, while decision curve analysis (DCA) quantified clinical net benefit. SHAP values were computed on a feature-level version of the combined model, containing the ten LASSO-retained radiomic features individually together with dichotomized admission D-dimer.

All statistical analyses were conducted using Python 3.8 and R software version 4.2.0, with significance threshold set at α = 0.05. To evaluate internal robustness beyond the single 7:3 split, we performed repeated stratified 10-fold cross-validation (50 repeats) and Harrell's optimism-corrected bootstrap (*B* = 1,000), with the complete feature-selection sequence re-executed within every resample. Out-of-sample performance was further examined within subgroups defined *post hoc* (age, sex, NIHSS score, DWI infarct volume) and across alternative cut-points for each stratifying variable, using a likelihood-ratio test for interaction between the model's linear predictor and the stratum indicator. As a sensitivity analysis for class imbalance, models were refit with class-weighted training and within-fold SMOTE oversampling. Model calibration was assessed by calibration curves, calibration slope and Brier score (Supplementary Figure S2, Supplementary Table S1).

### 2.6 Gene expression data acquisition and analysis

Two rat cerebral ischemia-related transcriptomic datasets, GSE61616 and GSE97537, were retrieved from the Gene Expression Omnibus (GEO) database. GSE61616 comprised brain tissue samples from 5 sham-operated controls and 5 middle cerebral artery occlusion (MCAO) model rats, while GSE97537 included samples from 5 sham-operated controls and 7 MCAO model rats. The gene list associated with the complement and coagulation cascades pathway was obtained from the KEGG pathway database (https://www.kegg.jp/). Differential expression of these candidate genes across both datasets was assessed using the GEO2R online analysis tool, with selection criteria defined as |log_2_fold change (FC)| > 1.0 and adjusted *P*-value (adj. *P*) < 0.05. Genes demonstrating significant differential expression in both datasets were identified as core candidate targets. Their potential associations with cerebral ischemia pathophysiology and prognosis were further explored through comprehensive literature review.

## 3 Results

### 3.1 Baseline characteristics

A total of 115 patients with acute ischemic stroke were enrolled and randomly allocated into training (*n* = 81) and testing (*n* = 34) cohorts at a 7:3 ratio. Baseline comparison revealed no significant differences between the two groups regarding age, sex, smoking history, alcohol consumption, hypertension, or other clinical characteristics (all *P* > 0.05, Table 1), confirming the validity of randomization and comparability of the datasets.

**Table 1:** Clinical baseline characteristics (*n* = 115).

Clinical characteristics	Training set (*n* = 81)	Testing set (*n* = 34)	*P*
Age^b^ [*M* (*P*_25_, *P*_75_), year]	70.00 (58.00, 77.00)	71.5 (61.00, 77.25)	0.481
Sex^a^, *n* (male/female)	57/24	20/14	0.230
Smoking history^a^, *n* (no/yes)	61/20	27/7	0.636
Alcohol history^a^, *n* (no/yes)	64/17	28/6	0.683
Hypertension^a^, *n* (no/yes)	17/64	9/25	0.521
Prior stroke^a^, *n* (no/yes)	66/15	24/10	0.196
LDL-C (mmol/L)^c^ (*x* ±*s*)	2.595 ± 0.797	2.630 ± 0.691	0.824
Creatinine (μmol/L)^c^ (*x* ±*s*)	88.078 ± 25.249	80.212 ± 27.371	0.146
HbA1c (%)^b^ [*M* (*P*_25_, *P*_75_)]	[7.300 (6.200, 9.150)]	[7.650 (6.275, 10.200)]	0.259
D-dimer^a^, *n* (Normal/Elevated)	43/38	17/17	0.762

a Categorical variables are presented as frequency (percentage) and compared using chi-square test or Fisher's exact test.

b Continuous variables are presented as median (interquartile range) and compared using Mann–Whitney *U* test.

c Continuous variables are presented as mean ± standard deviation and compared using independent samples *t*-test.

Patients were further stratified into favorable outcome (mRS 0–2) and unfavorable outcome (mRS 3–6) groups based on 90-day mRS scores, with comparative analyses performed separately in the training and testing cohorts. The distribution of outcomes was: training set 64 favorable/17 unfavorable (21.0% unfavorable); testing set 27/7 (20.6%); overall 91/24 (20.9%). In the training cohort, no significant differences were observed between the favorable and unfavorable outcome groups regarding traditional risk factors including age, sex, smoking history, alcohol consumption, and hypertension (all *P* > 0.05). Notably, D-dimer levels differed significantly between the two groups (*P* = 0.012), suggesting its potential role as an important clinical indicator for differentiating prognostic outcomes. In the testing cohort, although D-dimer levels did not reach statistical significance between groups (*P* = 0.398), the findings from the training cohort provided preliminary evidence supporting its value as a predictive factor. In the testing cohort, serum creatinine differed between outcome groups (*P* = 0.018); given the small number of events (*n* = 7) and the absence of any corresponding difference in the training cohort (*P* = 0.906), this isolated finding was not carried forward into model development.

These results indicate that D-dimer, serving as a biomarker reflecting coagulation status and thrombus burden, holds clinical relevance in prognostic assessment of acute ischemic stroke patients and warrants particular consideration in subsequent model development (Table 2).

**Table 2:** Comparison of clinical characteristics between training and testing sets (*n* = 115).

Clinical characteristics	Training set (***n*** = 81)			Testing set (***n*** = 34)		
	mRS 0–2 (***n*** = **64) |**	**mRS 3–6 (*****n*** = **17)**	***P***	**mRS 0–2 (*****n*** = **27)**	**mRS 3–6 (*****n*** = **7)**	***P***
Age^b^ [*M* (*P*_25_, *P*_75_), year]	70.00 (58.00, 77.75)	73.00 (63.50, 76.00)	0.741	72.00 (64.00, 80.00)	61.00 (60.00, 74.00)	0.151
Gendera *n* (male/female)	45/19	12/5	1.000	15/12	5/2	0.672
Smoking historya *n* (no/yes)	49/15	12/5	0.752	22/5	5/2	0.615
Alcohol history ^a^ *n* (no/yes)	52/12	12/5	0.334	22/5	6/1	1.000
Hypertension^a^ *n* (no/yes)	12/52	5/12	0.334	8/19	1/6	0.644
Prior stroke^a^ *n* (no/yes)	52/12	14/3	1.000	19/8	5/2	1.000
LDLC (mmol/L)^c^ (*x* ±*s*)	2.635 ± 0.855	2.446 ± 0.512	0.256	2.560 ± 0.618	2.900 ± 0.928	0.252
Creatinine (μmol/L)^c^ (*x* ±*s*)	88.259 ± 25.331	87.429 ± 25.714	0.906	74.488 ± 25.268	101.471 ± 25.808	0.018
HbA1c (%)^b^ [*M* (*P*_25_, *P*_75_)]	[7.450 (6.225, 9.175)]	[7.100 (6.050, 9.050)]	0.781	[7.300 (6.300, 10.200)]	[8.400 (6.200, 10.200)]	0.708
D-dimer^a^, *n* (Normal/Elevated)	39/25	4/13	0.012	15/12	2/5	0.398
Rad-score^c^ (*x* ±*s*)	−0.357 ± 0.670	0.899 ± 0.820	< 0.001	−0.236 ± 0.731	0.832 ± 0.863	< 0.001

^a^Categorical variables are presented as counts and compared using Fisher's exact test, which was applied to all categorical comparisons because the small number of unfavorable outcomes (17 in the training and 7 in the testing cohort) resulted in expected cell counts below 5 in most contingency tables. ^b^Continuous variables are presented as median (interquartile range) and compared using the Mann–Whitney *U* test. ^c^Continuous variables are presented as mean ± standard deviation and compared using Welch's *t*-test.

### 3.2 Feature extraction and model construction

**Figure 1:**
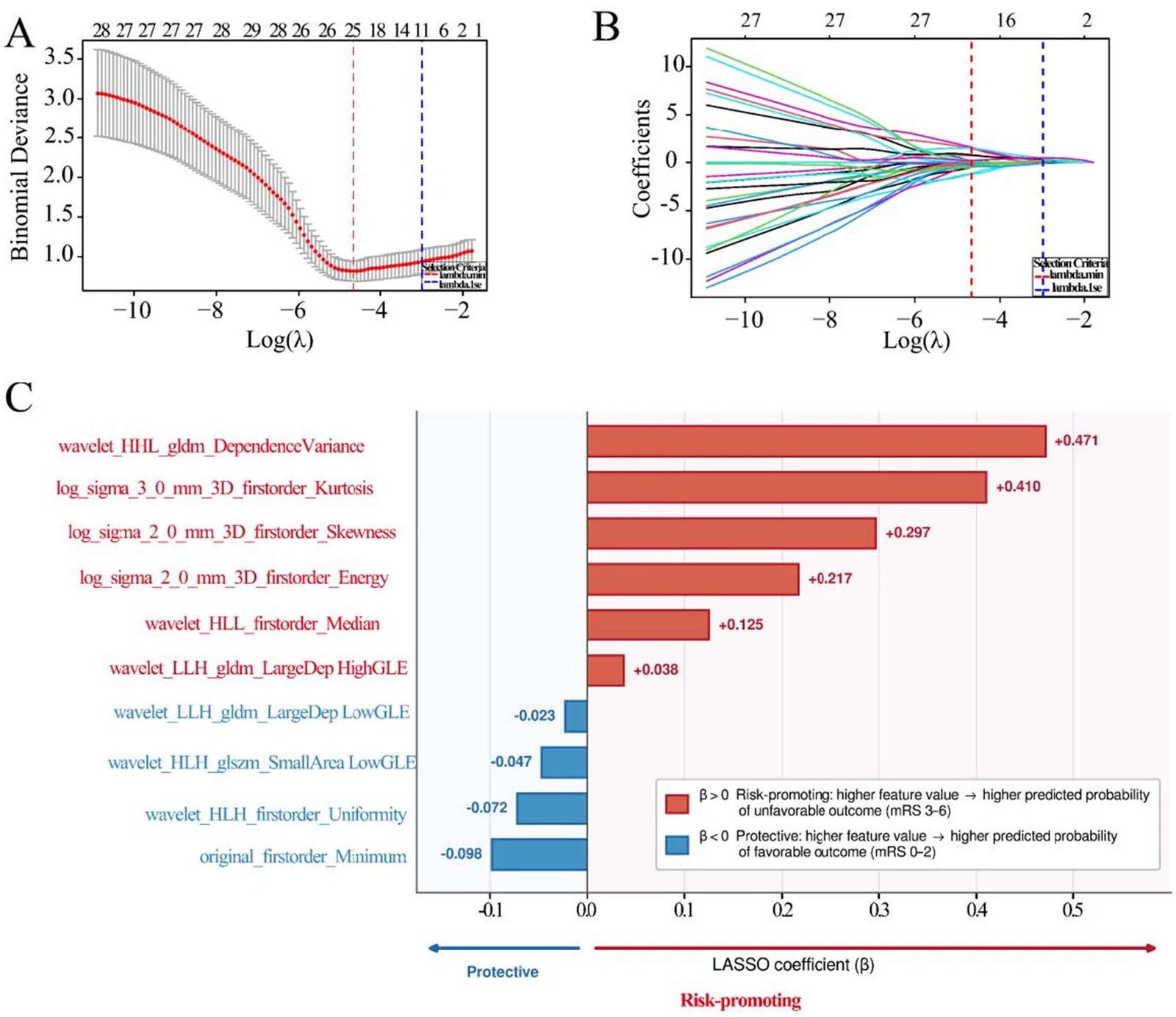
Radiomic feature selection using LASSO regression and key feature distribution. **(A)** Cross-validation error curve versus number of features; **(B)** LASSO coefficient paths; **(C)** Coefficients of the 10 radiomic features retained by LASSO regression. Features are color-coded by the direction of their association with the 90-day outcome: red bars (β > 0) denote risk-promoting features, for which higher values increase the predicted probability of an unfavorable outcome (mRS 3–6), whereas blue bars (β < 0) denote protective features, for which higher values increase the predicted probability of a favorable outcome (mRS 0–2).

A total of 1,024 radiomic features were extracted from the DWI images. After removing invalid features and retaining those with ICC > 0.75, 926 radiomic features remained. Following dimensionality reduction and feature selection (Figures 1A, B), 10 radiomic features were ultimately obtained (Figure 1C). These features were linearly weighted to calculate Rad-score, which were subsequently used to construct both traditional radiomic models and machine learning models via logistic regression.

**Figure 2:**
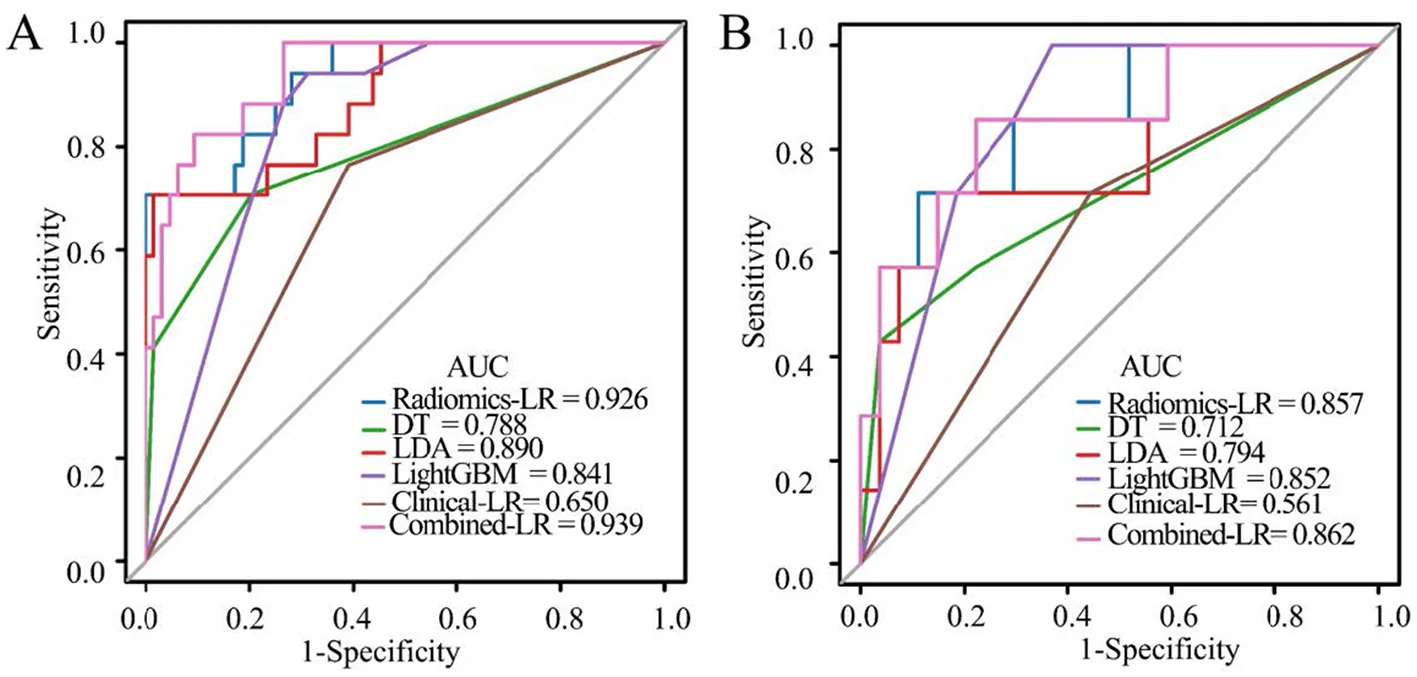
ROC curve comparison of prediction models in training **(A)** And testing **(B)** Cohorts.

Univariate logistic regression analysis in the training cohort revealed no significant differences for age, sex, hypertension, smoking history, or alcohol consumption, whereas D-dimer demonstrated statistical significance (*P* < 0.05). Multivariate logistic regression analysis identified D-dimer (*P* = 0.048, OR = 5.009, 95% CI: 1.011–24.814) and Rad-score (*P* < 0.001, OR = 10.646, 95% CI: 3.213–35.277) as statistically significant predictors. Accordingly, clinical and radiomic models were constructed using logistic regression. Collinearity analysis revealed no multicollinearity among independent risk factors (variance inflation factor < 10), indicating the absence of high correlation between variables, thereby supporting construction of the Combined-LR. ROC curve analysis demonstrated (Figures 2A, B; Table 3) that the Combined-LR achieved optimal predictive performance in both training and testing cohorts, with AUC values of 0.939 and 0.862, respectively. To assess robustness, we further performed repeated stratified 10-fold cross-validation (50 repeats) and optimism-corrected bootstrap (*B* = 1,000) with feature selection re-executed inside every fold; the optimism-corrected AUC of the Combined-LR was 0.838 (95% CI 0.765–0.895), 0.101 lower than the apparent estimate of 0.939 (Supplementary Table S1), supporting stable internal discrimination. Calibration curves for all six models in both cohorts are shown in Supplementary Figure S2. The Combined-LR was the best calibrated of the six, whereas LightGBM and the Decision Tree departed markedly from the diagonal in the low-probability range. The calibration curve of the Combined-LR was nevertheless flatter than the ideal diagonal in both cohorts: observed event proportions exceeded the predicted probabilities in the lower and middle range and fell below them at the upper end. This is the graphical signature of a calibration slope below unity, and it is confirmed numerically, the slope being 0.42 in the out-of-fold predictions of the training cohort and 0.19 for the frozen model in the testing cohort, with a corresponding out-of-fold Brier score of 0.153 (Supplementary Table S1). The testing-cohort value is estimated from 34 patients with 7 events and is therefore very imprecise. A slope below unity indicates that the predicted probabilities are more dispersed than the observed event rates warrant, so that shrinkage or recalibration would be required before the model is used to generate absolute risk estimates. The Hosmer–Lemeshow test was not used, because its power is negligible at this number of events and it is insensitive to the direction of miscalibration. The corresponding apparent-vs.-out-of-sample comparison is given in Supplementary Table S3. These out-of-sample predictions were then used for the *post hoc* subgroup analyses (age, sex, admission NIHSS and DWI infarct volume; Supplementary Table S2). No subgroup showed statistically significant heterogeneity of discrimination (*P* for interaction 0.537, 0.281, 0.165 and 0.140, respectively), and this conclusion was unchanged at every alternative cut-point examined and when infarct volume was modeled as a continuous variable (Supplementary Table S4). Discrimination was nevertheless numerically lower in patients with larger infarct volumes at every cut-point. Because a subgroup analysis performed in the patients used to fit the model would be optimistically biased, the corresponding in-sample estimates are given separately for comparison (Supplementary Table S5); the difference between the two indicates the magnitude of that bias. As a sensitivity analysis for class imbalance, all models were refitted with class-weighted training and within-fold synthetic minority over-sampling technique (SMOTE) oversampling using stratified 5-fold cross-validation with 20 repeats; the resulting test-cohort performance is compared with the primary analysis in Supplementary Table S6, and precision-recall and threshold-performance curves are shown in Supplementary Figure S1.

**Table 3:** Predictive performance of each model.

**Model**	Group	AUC (95% CI)	Accuracy	Sensitivity	Specificity
Radiomics-LR	Training set	0.926 (0.861–0.992)	0.938	0.706	1.000
	Testing set	0.857 (0.697–1.000)	0.853	0.714	0.889
DT Model	Training set	0.788 (0.658–0.917)	0.778	0.706	0.797
	Testing set	0.712 (0.479–0.944)	0.853	0.429	0.963
LDA Model	Training set	0.890 (0.799–0.980)	0.926	0.706	0.984
	Testing set	0.794 (0.588–1.000)	0.824	0.714	0.852
LightGBM Model	Training set	0.841 (0.759–0.922)	0.741	0.941	0.688
	Testing set	0.852 (0.733–0.971)	0.706	1.000	0.630
Clinical-LR	Training set	0.650 (0.523–0.777)	0.617	0.706	0.594
	Testing set	0.561 (0.357–0.765)	0.471	0.714	0.407
Combined-LR	Training set	0.939 (0.880–0.989)	0.778	1.000	0.719
	Testing set	0.862 (0.700–1.000)	0.794	0.857	0.778

### 3.3 Model comparison and interpretability analysis

Table 4 presents DeLong test results comparing AUC differences between models. The Combined-LR demonstrated statistically significant superiority over the clinical model (training cohort: *Z* = −4.101, *P* < 0.001; testing cohort: Z = −2.092, *P* = 0.036), indicating that the Combined-LR substantially outperformed the purely clinical parameter-based model. However, no significant differences were observed between the Combined-LR and most machine learning models (Radiomics-LR, LDA), suggesting comparable predictive performance among these radiomics-based models.

**Table 4:** DeLong test results for different models.

Model comparison	***Z***		***P***	
	Training set	Testing set	Training set	Testing set
Radiomics-LR vs. DT	1.942	1.338	0.052	0.181
Radiomics-LR vs. LDA	2.250	1.334	0.024	0.182
Radiomics-LR vs. LightGBM	1.919	0.069	0.055	0.945
DT vs. LDA	−1.312	−0.621	0.189	0.535
DT vs. LightGBM	−1.008	−1.443	0.314	0.149
LDA vs. LightGBM	0.914	−0.541	0.361	0.588
Radiomics-LR vs. Clinical-LR	3.322	2.063	< 0.001	0.039
Radiomics-LR vs. Combined-LR	−0.661	−0.338	0.509	0.735
DT vs. Clinical-LR	0.980	0.494	0.327	0.622
DT vs. Combined-LR	−2.122	−1.432	0.034	0.152
LDA vs. Clinical-LR	2.514	1.152	0.012	0.249
LDA vs. Combined-LR	−1.558	−1.209	0.119	0.227
LightGBM vs. Clinical-LR	1.951	1.868	0.051	0.062
LightGBM vs. Combined-LR	−2.209	−0.137	0.027	0.891
Clinical-LR vs. Combined-LR	−4.101	−2.092	< 0.001	0.036

**Figure 3:**
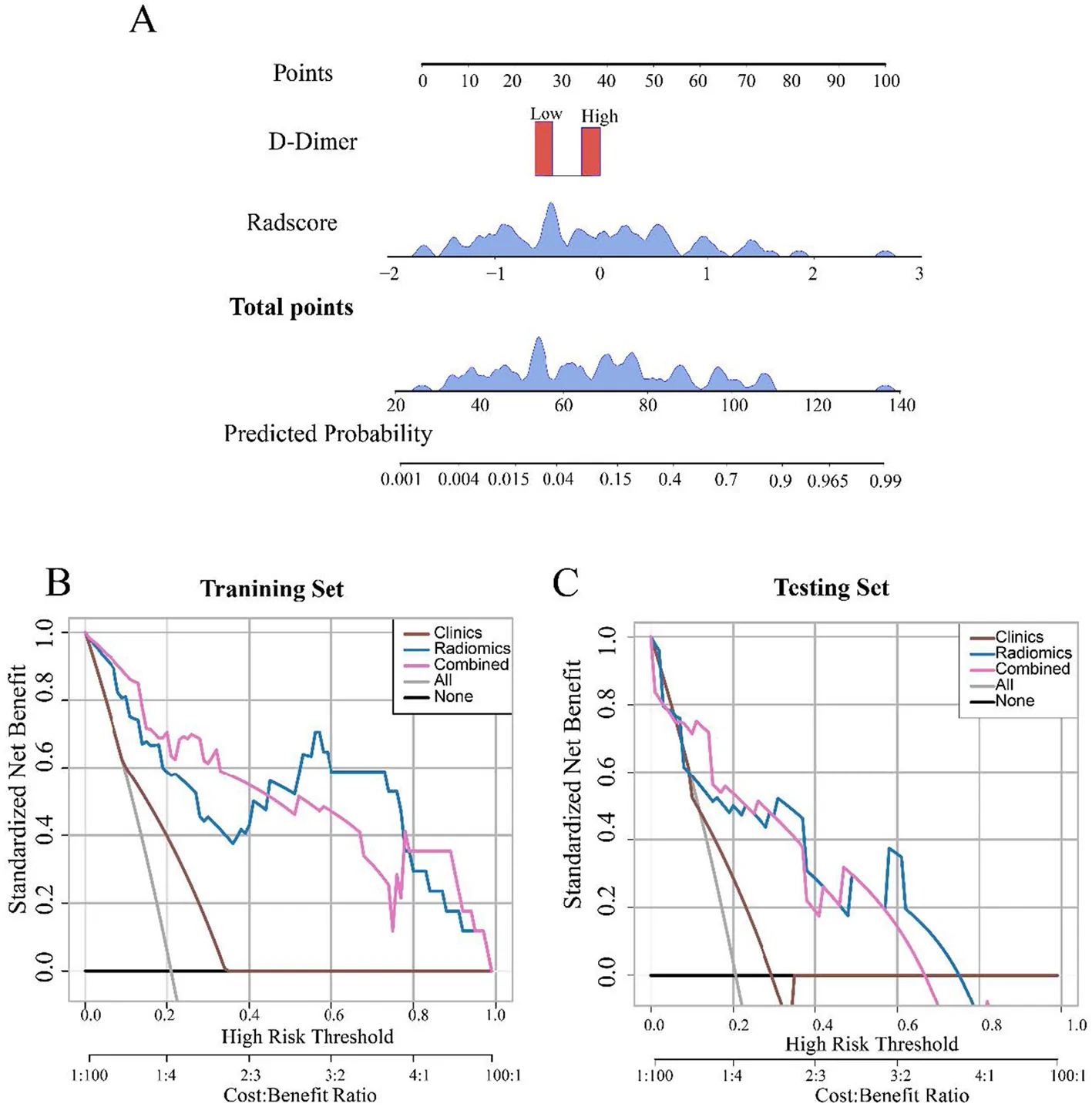
Clinical application tool and performance evaluation of the combined-LR **(A)** Prediction nomogram; **(B)** Decision curve analysis in training set; **(C)** Decision curve analysis in test set.

Figure 3A illustrates the nomogram constructed based on the Combined-LR, integrating two key predictive factors: D-dimer level and radiomic score. The nomogram employs a point-based scoring system, enabling clinicians to rapidly calculate total points based on patient-specific parameters and derive the predicted probability of unfavorable 90-day functional outcome, thereby providing an intuitive quantitative tool for clinical decision-making. Decision curve analysis (Figures 3B, C) demonstrated that the Combined-LR achieved the highest clinical net benefit across most threshold probability ranges, confirming its practical utility and decision support capability in clinical prognostication. Within this development cohort, the Combined-LR therefore offered the most favorable balance of net benefit among the models compared; whether this translates into practical value in other settings remains to be established by external validation.

**Figure 4:**
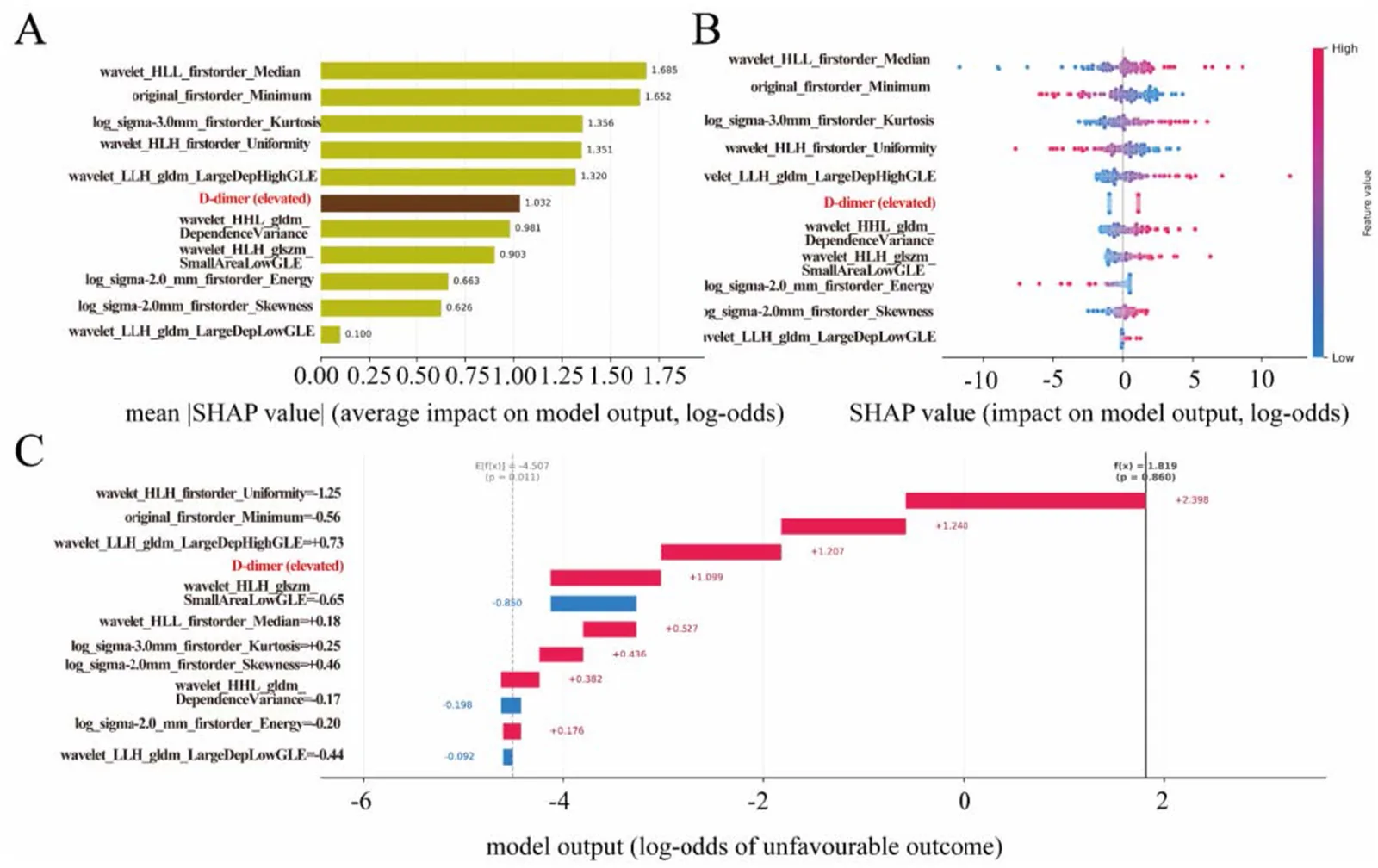
SHAP-based interpretability analysis. Panels **(A–C)** refer to a feature-level logistic model containing the ten LASSO-retained radiomic features together with dichotomized admission D-dimer. A. Global feature importance ranking. **(B)** Beeswarm plot showing the distribution of SHAP values for each feature. Each point represents a patient; the color indicates the feature value (red for high, blue for low), and the position on the x-axis indicates the impact on the model prediction (positive values increase the risk of unfavorable outcome). **(C)** Waterfall plot explaining the prediction for an individual representative patient. It illustrates how each feature contributes positively (red) or negatively (blue) to shift the model output from the base value (expected value) to the final prediction.

To elucidate the prediction mechanisms of machine learning models and enhance clinical credibility, SHAP analysis was employed for interpretability assessment of the feature-level expansion of the Combined-LR. Figure 4A illustrates the global ranking of feature importance based on the mean absolute SHAP values. Contrary to previous unimodal analyses, the Combined-LR highlights that wavelet-HLL_firstorder_Median (mean |SHAP| = 1.685) and original_firstorder_Minimum (mean |SHAP| = 1.652) are the top two most influential predictors. These first-order statistical features reflect the central tendency and the lowest signal intensity within the lesion volume, respectively, suggesting that the overall texture baseline and potential necrotic cores are critical drivers of the model's decision. Notably, the clinical biomarker D-dimer (elevated) ranks sixth (mean |SHAP| = 1.032), surpassing several complex radiomic signatures (e.g., DependenceVariance). This indicates that, despite the high dimensionality of the imaging data, coagulation-fibrinolysis status contributed an axis of risk that the radiomic features did not reproduce in this cohort.

Figure 4B presents the beeswarm plot, detailing the distribution of SHAP values for each feature across the dataset. The plot reveals distinct directional impacts: For original_firstorder_Minimum, higher feature values (red dots) predominantly cluster on the negative side of the SHAP axis while lower values cluster on the positive side, indicating that a higher minimum signal intensity within the lesion shifts the prediction toward a favorable outcome. This direction is consistent with the negative LASSO coefficient of this feature (β = −0.098, Figure 1C). Crucially, for D-dimer, the plot demonstrates a clear positive correlation: elevated levels (red dots) consistently align with positive SHAP values, driving the model output toward a higher probability of poor prognosis. This visualizes the synergistic effect where both specific imaging phenotypes and systemic biomarkers contribute additively to the risk stratification.

Figure 4C illustrates the individual prediction process for a specific patient using a waterfall plot, bridging the gap between population-level statistics and patient-specific interpretation. Starting from the model's baseline output *E*[*f* (*x*)] = −4.507, features cumulatively shift the prediction toward the final outcome. In this high-risk case, the model predicts an unfavorable 90-day functional outcome with a probability of 86.0% [*f* (*x*) = 1.819]. Notably, the clinical predictor D-dimer (elevated) acts as a significant risk driver, contributing a positive SHAP value of +1.099, which substantially pushes the prediction toward the unfavorable class alongside top radiomic features. In this individual prediction, the largest radiomic contribution comes from wavelet-HLH_firstorder_Uniformity, contributing +2.398 (Figure 4C). In this patient the standardized value of this feature is −1.25, i.e., well below the cohort mean; because the feature carries a negative model coefficient (β = −0.072, Figure 1C), a low value shifts the prediction upward. Low texture uniformity within the lesion is therefore the single largest contributor to this patient's predicted risk. The next two contributions come from original_firstorder_Minimum (value −0.56, contribution +1.240) and wavelet-LLH_gldm_LargeDepHighGLE (value +0.73, contribution +1.207), both of which also push the prediction upward in this individual. This visualization illustrates how the Combined-LR combines a clinical biomarker with imaging phenotypes when stratifying an individual patient's risk.

### 3.4 Differential expression analysis of key genes in the complement-coagulation pathway

**Figure 5:**
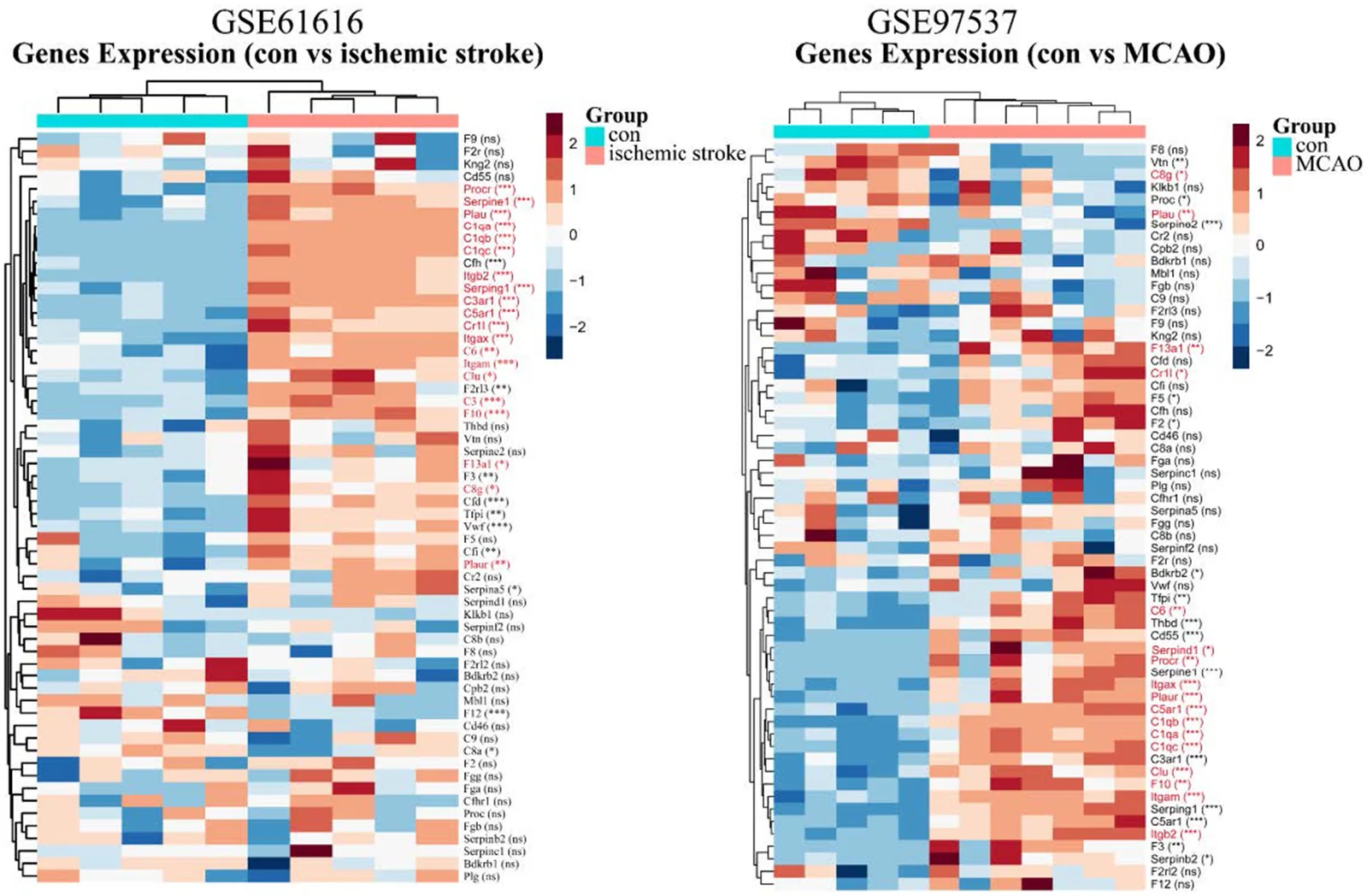
Differential expression analysis of genes related to complement and coagulation cascade pathway in rat brain with ischemic stroke.

To generate candidate upstream mechanisms for the D-dimer signal, we performed an exploratory transcriptomic screen in two independent rat MCAO datasets. This analysis is not paired with human plasma measurements and does not establish causality; it identifies pathway-level candidates for future validation. Genes associated with the complement and coagulation cascades pathway exhibited significant differential expression in brain tissue from MCAO model rats across both databases (Figure 5). Across two independent datasets, we observed significant upregulation of *Serpine1* (PAI-1, log_2_FC = 2.3, *P* < 0.001), *C5ar1* (log_2_FC = 1.8, *P* < 0.01), and *Itgam* (CD11b, log_2_FC = 2.1, *P* < 0.001), genes representing three distinct pathological processes: fibrinolytic inhibition, complement activation, and neutrophil infiltration, respectively.

PAI-1 is the principal endogenous inhibitor of tissue plasminogen activator, and its overexpression suppresses plasminogen activation so that fibrin already formed is less readily lysed, a state that has been associated with resistance to thrombolysis (13). CD11b-positive neutrophils contribute to thrombus growth and to activation of coagulation through the release of neutrophil extracellular traps (14). C5aR1 mediates complement C5a signaling and has been implicated in thrombus propagation and blood-brain barrier disruption (15). These transcriptomic findings are compatible with the hypothesis that an elevated admission D-dimer reflects coordinated activation across these pathways, but the datasets are derived from rat brain parenchyma and are entirely independent of the clinical cohort. They are therefore put forward as candidate upstream mechanisms to be tested in studies in which these markers and D-dimer are measured in the same patients, and not as an explanation of the association reported here.

## 4 Discussion

Among patients treated with intravenous thrombolysis, a substantial proportion still reach 90 days with moderate-to-severe disability (16), and identifying them early, at a point at which monitoring and rehabilitation planning can still be altered, is of direct clinical consequence (17). In the present cohort, DWI radiomic features alone supported reasonable discrimination (Radiomics-LR: AUC 0.926 in the training and 0.857 in the testing cohort), and the addition of admission D-dimer improved it further (Combined-LR: 0.939 and 0.862). These are apparent estimates. After correction for optimism the same model gave an AUC of 0.838 (95% CI 0.765–0.895) by Harrell's bootstrap and 0.772 (SD 0.017) by repeated stratified 10-fold cross-validation with the complete feature-selection sequence re-executed inside every fold (Supplementary Table S1).

Three properties of D-dimer are conflated in the existing literature, and in our own earlier draft, into a single circular statement, that D-dimer predicts poor outcome because patients with poor outcome have high D-dimer. In the following three subsections we separate them: the amount of clot present before treatment (3.1), the susceptibility of that clot to lysis by tPA (3.2), and the upstream coagulation–inflammation programmes that plausibly generate both (3.3). Only the first two are supported by data from the present cohort; the third is an exploratory hypothesis derived from an independent animal dataset.

### 4.1 D-dimer as an index of thrombus burden

Elevated admission D-dimer was considerably more common among patients with an unfavorable 90-day outcome than among those with a favorable one (75.0% vs. 40.7%), and D-dimer remained an independent predictor in the multivariable model after adjustment for the radiomic signature (OR 5.009, 95% CI 1.011–24.814), consistent with previous reports in thrombolysed cohorts (18).

D-dimer is a degradation product of cross-linked fibrin, and its plasma concentration therefore scales with the quantity of fibrin that has been generated and subsequently lysed (19). Read this way, a higher admission value is most parsimoniously interpreted as a larger intravascular thrombus and, by extension, a larger volume of tissue at risk; a comparable relationship between D-dimer and thrombus mass has been documented in other thrombo-embolic conditions (20). This interpretation is deliberately restricted. It relates D-dimer to the size of the occluding clot at a single time point before treatment, and it makes no claim about the biological response that follows, which is the subject of the two subsections that follow.

### 4.2 D-dimer and resistance to tPA

Thrombus burden alone does not account for the whole of the association, because clots of equal size are not equally lysable. Thrombi retrieved from patients with acute ischemic stroke differ substantially in composition, and fibrin-rich and platelet-rich material is considerably less susceptible to plasminogen-activator-mediated lysis than erythrocyte-rich material; platelet-rich thrombi in particular show tPA resistance that can be overcome only by targeting non-fibrin components (21, 22). Neutrophil extracellular traps (NETs) provide a further non-fibrin scaffold, and their content in retrieved stroke thrombi is inversely related to the efficacy of tPA-induced thrombolysis (14).

A given admission D-dimer concentration therefore carries information about how readily a clot will lyse as well as about how much clot is present. This offers one account of why D-dimer retained independent predictive value in the Combined-LR model alongside imaging descriptors of lesion size and texture: the imaging features characterize the infarct that has already occurred, whereas D-dimer additionally indexes a property of the occluding material that determines whether reperfusion will be achieved. We note that this remains an inference from the literature rather than a measurement in our cohort, since thrombus composition was not available for our patients.

### 4.3 Coagulation–inflammation crosstalk: candidate upstream programmes

Neither thrombus burden nor clot composition specifies the upstream programmes that generate them, and previous work has largely treated D-dimer as an isolated measurement rather than as the output of a regulated balance between coagulation and fibrinolysis. To generate hypotheses about that balance, we screened two independent rat MCAO transcriptomic datasets and identified *Serpine1* (PAI-1), *Itgam* (CD11b) and *C5ar1* as differentially expressed within the complement and coagulation cascades pathway, corresponding respectively to fibrinolytic inhibition, neutrophil-mediated immunothrombosis and complement–coagulation crosstalk (23–25).

The evidence bridging each of these genes to a human plasma measurement is of unequal strength, and we set the three out separately rather than presenting them as equivalent. For PAI-1 the bridge is the most direct: PAI-1 is the principal endogenous inhibitor of tPA, and higher circulating levels have been associated with thrombolysis failure in stroke patients (13). For CD11b the bridge is indirect but supported by human thrombus material, in which NET content impairs tPA-induced thrombolysis and relates to stroke severity (14). For C5ar1 the bridge is the weakest, and we state this explicitly: plasma C5a in humans rises with a delay of roughly 1–2 weeks after onset (15), which is not compatible with a contribution to a D-dimer value measured on admission. We therefore regard C5ar1 as a candidate mechanism for subsequent secondary injury rather than for the admission biomarker, and we do not offer it as an explanation of the association reported here.

Taken together, these observations suggest, but do not establish, that an elevated admission D-dimer may reflect a dual imbalance of hyperactivated coagulation and suppressed fibrinolysis rather than excessive thrombosis alone (26). Such a framework would offer one explanation for the limited efficacy of antiplatelet or anticoagulant monotherapy, which addresses neither upstream immune-cell infiltration nor the endogenous fibrinolytic capacity suppressed by PAI-1 (27). The limits of this inference should be stated plainly. The transcriptomic datasets derive from rat brain parenchyma rather than human plasma; our clinical cohort is entirely independent of them; and no molecular measurements were available in our patients. Confirming any of these links would require PAI-1, C5a and NET markers to be measured in the same patients in whom D-dimer and 90-day outcome are recorded, which is the design we intend for the prospective cohort now being assembled.

The models built on DWI radiomic features outperformed the Clinical-LR model, which is consistent with the physical basis of the sequence. DWI is sensitive to the restriction of water diffusion produced by cytotoxic oedema, so that the ischemic lesion is delineated earlier and more sharply than on other sequences, and its texture carries information about lesion heterogeneity that a small set of admission variables cannot represent. Previous work has reported comparable value for DWI radiomics in predicting long-term outcome after stroke (28), and our results are concordant.

Among the six models compared, the two radiomics-based logistic-regression models performed best. Radiomics-LR is a generalized linear model with few effective parameters, which is an advantage rather than a limitation at this sample size; inadequate sample size relative to model complexity is a recognized and pervasive problem in radiomics prediction studies (29), and flexible learners are the first to suffer from it. LDA performed slightly less well, which is unsurprising given that it assumes normally distributed features with equal class covariances (30), assumptions that are unlikely to hold with 17 unfavorable outcomes in the training cohort. The decision tree performed least well, reflecting the instability of a single unpruned partition at this sample size.

LightGBM produced the most asymmetric profile: sensitivity 1.000 with specificity 0.630 in the testing cohort. This profile is the expected consequence of fitting a gradient-boosting ensemble to an imbalanced sample at a fixed 0.5 probability threshold. The boosting objective is dominated by the 64 favorable outcomes in the training cohort, and with only 17 unfavorable events the predicted-probability distributions of the two classes overlap substantially, so that a threshold placed at 0.5 falls where a large proportion of favorable cases still receive predictions above it. Consistent with this account, refitting with class weighting and within-fold SMOTE raised specificity from 0.630 to 0.889 (Supplementary Table S6). The costs of the two error types in post-thrombolysis triage are genuinely asymmetric, and this deserves to be stated before the model is dismissed. A false positive leads to closer neurological monitoring, earlier rehabilitation referral and more intensive secondary prevention, interventions that carry limited risk and, in many cases, some benefit even for a patient who would have recovered without them. A false negative means that a patient with a high probability of severe disability is not identified and the opportunity for early intensified care is lost. On that reasoning an operating point favoring sensitivity is defensible in principle. In practice, however, a specificity of 0.630 means that 10 of the 27 patients with a favorable outcome in the testing cohort would have been flagged as high risk; at that rate the model adds little beyond the base rate, and repeated false alarms would erode clinician confidence. We therefore do not put LightGBM forward as the model to be used. The Combined-LR model, with a more balanced profile (sensitivity 0.857, specificity 0.778) and the highest net benefit across the clinically relevant threshold range in decision curve analysis, is the model we take forward, subject to the external validation described below. Threshold performance curves are provided in Supplementary Figure S1 so that readers may select an operating point appropriate to their own setting.

SHAP analysis was applied to make the model's use of its inputs inspectable. Two first-order intensity descriptors dominated the ranking: wavelet-HLL first-order Median (mean |SHAP| = 1.685), which reflects the central tendency of signal intensity within the lesion after HLL wavelet decomposition, and original first-order Minimum (mean |SHAP| = 1.652), which captures the lowest signal within the lesion and plausibly indexes necrotic core involvement. The two act in opposite directions, higher values of the former shifting predictions toward an unfavorable outcome and higher values of the latter toward a favorable one. Admission D-dimer ranked sixth of eleven features (mean |SHAP| = 1.032), above several higher-order texture descriptors, indicating that it contributes an axis of risk that the imaging features do not reproduce. The beeswarm plot shows a monotonic positive relationship between elevated D-dimer and model output, concordant with the univariable comparison, and the waterfall example illustrates how the contributions accumulate from the population baseline to an individual prediction.

This study has several limitations. First, as a single-center retrospective study using a single Philips 3.0T platform without external validation, the model's generalizability is limited due to the known sensitivity of radiomic features to scanner and protocol variations. Second, while internal robustness was assessed via out-of-sample predictions across subgroups (Supplementary Table S2), the analyses were underpowered (total 24 events). The strata defined by low NIHSS ( ≤ 5) and small infarct volume ( ≤ 2.42 ml) contained only 4 and 6 events respectively, so that the high AUC values observed in these two strata (0.924 and 0.846) rest on a handful of outcomes, carry very wide confidence intervals, and should not be read as evidence of strong performance in mild stroke. Calibration was moreover imprecisely estimated, particularly in the testing cohort, where only 7 unfavorable outcomes were available, and the calibration slope fell below unity; external recalibration is therefore required before the nomogram is used to generate absolute risk estimates rather than a ranking of risk. Notably, discrimination was lower in patients with larger infarct volumes, and the Rad-score correlated strongly with lesion volume (Spearman rho = 0.74), suggesting the model's performance may partly reflect lesion size rather than adding independent prognostic value in severe cases. Third, class imbalance (20.9% unfavorable outcomes) affected the operating characteristics of the models. Refitting with class weighting and within-fold SMOTE raised LightGBM specificity from 0.630 to 0.889 at the cost of sensitivity (1.000–0.857), which supports imbalance rather than intrinsic model failure as the explanation for its original profile. The same procedure did not improve the logistic-regression models: it lowered the specificity of Radiomics-LR (0.889–0.481) and of LDA (0.852–0.481) and the AUC of the Combined-LR (0.862–0.788). Resampling therefore alters where the decision threshold falls rather than improving discrimination, and the unresampled models were retained as the primary analysis (Supplementary Table S6). Fourth, the lack of standardized vascular imaging precluded reliable assessment of occlusion sites; DWI infarct volume was used as a proxy for stroke burden. Fifth, manual ROI delineation introduces potential observer bias, though reproducibility was acceptable; automated segmentation is preferred for future studies. Sixth, the reliance on DWI alone excludes potential information from apparent diffusion coefficient, perfusion, or susceptibility-weighted imaging sequences. Finally, the transcriptomic component is exploratory, derived from independent rat datasets rather than patient samples, serving only to identify candidate pathways for future validation. In addition, although admission NIHSS was recorded, it was not entered into the clinical model, which contained D-dimer alone; the comparison between the combined and clinical models therefore does not establish superiority over a conventional severity-based assessment, and NIHSS was used here only as a stratifying variable.

Within this single-center development cohort, a logistic model combining DWI radiomic features with admission D-dimer achieved discrimination adequate for internal risk stratification of 90-day functional outcome after intravenous thrombolysis, outperformed a model built on clinical variables alone, and provided the highest net benefit in decision curve analysis. SHAP analysis rendered its use of imaging and clinical inputs inspectable at both the cohort and the individual level. These findings are exploratory. External multicenter validation on scanners and protocols other than our own is a prerequisite for clinical use, and the rat transcriptomic analysis should be read as generating candidate upstream pathways rather than as mechanistic evidence for the prognostic value of D-dimer in humans.

## Data Availability

The datasets presented in this study can be found in online repositories. The names of the repository/repositories and accession number(s) can be found below: https://www.ncbi.nlm.nih.gov/geo/, GSE97537, GSE61616.
