# Supplementary material for: Combined clinical and DWI radiomics model for predicting 90-day functional outcomes after intravenous thrombolysis in acute ischemic stroke patients
Source: Front Neurol. 2026 Aug 28;17:1847290. doi: 10.3389/fneur.2026.1847290 (PMC13561888; doi:10.3389/fneur.2026.1847290)
Supplement: Supplementary file 1 [file Data_Sheet_1.docx]

# Supplementary Materials

**Table S1. Internal validation of the combined clinical–radiomics model.**

| **Validation procedure** | **AUC** | **95% interval** |  |
| --- | --- | --- | --- |
| Reported 7:3 split — training set (n = 81) | 0.939 | — |  |
| Reported 7:3 split — testing set (n = 34) | 0.862 | — |  |
| Repeated stratified 10-fold cross-validation (50 repeats) | 0.772 (SD 0.017) | 0.735–0.802 |  |
| Optimism-corrected bootstrap (B = 1000) | 0.838 | 0.765–0.895 |  |
| Calibration slope (out-of-fold predictions, training cohort) | 0.42 | — |  |
| Calibration slope (frozen model, testing set) | 0.19 | — |  |
| Brier score (training cohort) | 0.153 | — |  |

All resampling procedures repeat the complete modelling sequence, standardization, t-test filtering, correlation pruning, mRMR, LASSO and logistic regression, within each resample, so that no information from held-out patients enters feature selection. Estimates are therefore lower than, and not directly comparable with, apparent performance in a single split.

**Table S2. Out-of-sample performance of the combined radiomics–clinical model within post hoc subgroups (all 115 patients, no patient contributing to the model that predicts them).**

| **Subgroup** | **Stratum** | **n** | **Events** | **AUC (95% CI)** | **Brier** | **P for interaction** |
| --- | --- | --- | --- | --- | --- | --- |
| Overall | All patients | 115 | 24 | 0.789 (0.702–0.874) | 0.136 | — |
| Age | ≤71 years | 60 | 13 | 0.786 (0.651–0.894) | 0.142 |  |
|  | ＞71 years | 55 | 11 | 0.818 (0.688–0.930) | 0.130 | 0.537 |
| Sex | Female | 38 | 7 | 0.682 (0.484–0.853) | 0.150 |  |
|  | Male | 77 | 17 | 0.831 (0.718–0.924) | 0.129 | 0.281 |
| NIHSS | ≤5 | 63 | 4 | 0.924 (0.830–0.992) | 0.074 |  |
|  | ＞5 | 52 | 20 | 0.797 (0.664–0.911) | 0.212 | 0.165 |
| DWI infarct volume | ≤2.42 mL | 58 | 6 | 0.846 (0.727–0.949) | 0.074 |  |
|  | ＞2.42 mL | 57 | 18 | 0.677 (0.524–0.823) | 0.200 | 0.140 |

AUC, area under the receiver operating characteristic curve; NIHSS, National Institutes of Health Stroke Scale. P for interaction is from a likelihood-ratio test of linear predictor by subgroup.

**Table S3. Apparent versus out-of-sample discrimination (optimism).**

| **Cohort** | **Apparent AUC** | **Out-of-sample AUC** | **Optimism** |
| --- | --- | --- | --- |
| Training cohort (n = 81) | 0.939 | 0.766 | 0.173 |
| Testing cohort (n = 34) | — | 0.862 | — |
| Full cohort (n = 115) | 0.865 | 0.789 | 0.076 |

Apparent AUC is calculated using the fitted model in the same patients used to fit it. Out-of-sample AUC uses repeated cross-validated predictions in the training cohort and the frozen model in the testing cohort. Optimism is the difference between the two. Out-of-sample AUC is computed on the pooled out-of-fold predictions and therefore differs slightly from the mean of fold-wise AUCs reported in Table S1.

**Table S4. Sensitivity of the subgroup findings to the choice of cut-point (out-of-sample predictions).**

| **Cut-point** | **n (low)** | **n (high)** | **AUC low** | **AUC high** | **ΔAUC (95% CI)** | **P-int** |
| --- | --- | --- | --- | --- | --- | --- |
| Age ＞65 years | 40 | 75 | 0.805 | 0.797 | −0.008 (−0.185, +0.176) | 0.486 |
| Age ＞75 years | 78 | 37 | 0.761 | 0.898 | +0.137 (−0.054, +0.286) | 0.131 |
| NIHSS ≥8 | 72 | 43 | 0.926 | 0.778 | −0.148 (−0.325, +0.018) | 0.129 |
| NIHSS ＞4 | 55 | 60 | 0.906 | 0.789 | −0.116 (−0.278, +0.073) | 0.385 |
| Volume ≥5 mL | 73 | 42 | 0.816 | 0.681 | −0.135 (−0.337, +0.075) | 0.408 |
| Volume ≥10 mL | 91 | 24 | 0.800 | 0.593 | −0.207 (−0.460, +0.069) | 0.273 |
| Volume, upper tertile | 77 | 38 | 0.834 | 0.700 | −0.134 (−0.350, +0.070) | 0.382 |
| Volume, continuous log₁₀(mL) | — | — | — | — | — | 0.142 |

ΔAUC is the difference between the high and low strata, with 95% confidence intervals from bootstrap resampling. P-int is the likelihood-ratio test for interaction between the model's linear predictor and the stratum indicator.

**Table S5. Corresponding analysis using the frozen model applied to all 115 patients, shown for comparison.**

| **Subgroup** | **Stratum** | **n** | **Events** | **AUC (95% CI)** | **Sens.** | **Spec.** |
| --- | --- | --- | --- | --- | --- | --- |
| Overall | All patients | 115 | 24 | 0.865 (0.790–0.928) | 0.792 | 0.835 |
| Age | ≤71 years | 60 | 13 | 0.869 (0.771–0.948) | 0.692 | 0.872 |
|  | ＞71 years | 55 | 11 | 0.870 (0.756–0.959) | 0.909 | 0.795 |
| Sex | Female | 38 | 7 | 0.779 (0.599–0.917) | 0.714 | 0.774 |
|  | Male | 77 | 17 | 0.895 (0.808–0.964) | 0.824 | 0.867 |
| NIHSS | ≤5 | 63 | 4 | 0.953 (0.881–1.000) | 1.000 | 0.831 |
|  | ＞5 | 52 | 20 | 0.866 (0.756–0.955) | 0.750 | 0.844 |
| DWI infarct volume | ≤2.42 mL | 58 | 6 | 0.965 (0.894–1.000) | 0.833 | 0.981 |
|  | ＞2.42 mL | 57 | 18 | 0.756 (0.615–0.883) | 0.778 | 0.641 |

In this analysis the 81 patients of the training cohort receive predictions from the model that was fitted using them, and the resulting estimates are therefore optimistically biased; the table is provided so that the magnitude and direction of that bias can be judged directly against Table S2. Note in particular that the two smallest strata (NIHSS ≤5 and volume ≤2.42 mL) show the largest inflation. Table S2 constitutes the primary analysis.

**Table S**6**.** Test-cohort performance of all six models in the original main analysis (Table 3 of the manuscript) and in the class-imbalance sensitivity analysis.

| **Model** | **Analysis** | **AUC (95% CI)** | **PR-AUC** | **Accuracy** | **Sensitivity** | **Specificity** |
| --- | --- | --- | --- | --- | --- | --- |
| **Radiomics-LR** | Original | 0.857 (0.697-1.000) | — | 0.853 | 0.714 | 0.889 |
|  | Sensitivity (SMOTE + class-weighted) | 0.767 (0.550-0.939) | 0.463 (0.198-0.850) | 0.588 | 1.000 | 0.481 |
| **DT** | Original | 0.712 (0.479-0.944) | — | 0.853 | 0.429 | 0.963 |
|  | Sensitivity (SMOTE + class-weighted) | 0.741 (0.534-0.911) | 0.408 (0.154-0.704) | 0.765 | 0.714 | 0.778 |
| **LDA** | Original | 0.794 (0.588-1.000) | — | 0.824 | 0.714 | 0.852 |
|  | Sensitivity (SMOTE + class-weighted) | 0.762 (0.550-0.925) | 0.445 (0.186-0.821) | 0.588 | 1.000 | 0.481 |
| **LightGBM** | Original | 0.852 (0.733-0.971) | — | 0.706 | 1.000 | 0.630 |
|  | Sensitivity (SMOTE + class-weighted) | 0.894 (0.710-1.000) | 0.804 (0.491-1.000) | 0.882 | 0.857 | 0.889 |
| **Clinical-LR** | Original | 0.561 (0.357-0.765) | — | 0.471 | 0.714 | 0.407 |
|  | Sensitivity (SMOTE + class-weighted) | 0.635 (0.421-0.827) | 0.269 (0.103-0.500) | 0.588 | 0.714 | 0.556 |
| **Combined-LR** | Original | 0.862 (0.700-1.000) | — | 0.794 | 0.857 | 0.778 |
|  | Sensitivity (SMOTE + class-weighted) | 0.788 (0.579-0.958) | 0.550 (0.217-0.887) | 0.853 | 0.571 | 0.926 |

The class-imbalance sensitivity analysis (Table S6, Figure S1) used stratified 5-fold cross-validation with 20 repeats, which differs from the 10-fold, 50-repeat scheme used for the primary internal validation in Table S1; the two analyses address different questions and are not directly comparable.

**Figure S1.** (A, B) Precision-recall curves on the test cohort and on the pooled cross-validation folds of the training cohort. The dashed horizontal line marks the positive-class prevalence, i.e. the PR curve of a non-informative classifier. (C) F1 as a function of the decision threshold on the test cohort for all six models. (D–F) Threshold–performance curves for Radiomics-LR, LightGBM and the Combined model: sensitivity, specificity, precision and F1 as a function of the decision threshold, with the Youden-index-optimal threshold marked by a dashed vertical line.

**Figure S2.** Calibration curves for all six prediction models in the training and testing cohorts. The x-axis is the predicted probability of an unfavorable 90-day outcome and the y-axis the observed event proportion within each group of predicted risk; the diagonal represents perfect calibration. Curves lying below the diagonal indicate predicted risks that exceed observed rates. Calibration slopes and Brier scores for the Combined-LR are given in Supplementary Table S1.
